# Inspection and Reconstruction of Metal-Roof Deformation under Wind Pressure Based on Bend Sensors

**DOI:** 10.3390/s17051054

**Published:** 2017-05-06

**Authors:** Liman Yang, Langfu Cui, Yunhua Li, Chao An

**Affiliations:** School of Automation Science and Electrical Engineering, Beihang University, XueYuan Road No. 37, HaiDian District, Beijing 100191, China; cuilangfu@buaa.edu.cn (L.C.); ac2005@sina.com (C.A.)

**Keywords:** metal roof, fast reconstruction model, bend sensor, elastic thin plate

## Abstract

Metal roof sheathings are widely employed in large-span buildings because of their light weight, high strength and corrosion resistance. However, their severe working environment may lead to deformation, leakage and wind-lift, etc. Thus, predicting these damages in advance and taking maintenance measures accordingly has become important to avoid economic losses and personal injuries. Conventionally, the health monitoring of metal roofs mainly relies on manual inspection, which unavoidably compromises the working efficiency and cannot diagnose and predict possible failures in time. Thus, we proposed a novel damage monitoring scheme implemented by laying bend sensors on vital points of metal roofs to precisely monitor the deformation in real time. A fast reconstruction model based on improved Levy-type solution is established to estimate the overall deflection distribution from the measured data. A standing seam metal roof under wind pressure is modeled as an elastic thin plate with a uniform load and symmetrical boundaries. The superposition method and Levy solution are adopted to obtain the analytical model that can converge quickly through simplifying an infinite series. The truncation error of this model is further analyzed. Simulation and experiments are carried out. They show that the proposed model is in reasonable agreement with the experimental results.

## 1. Introduction

**Figure 1:**
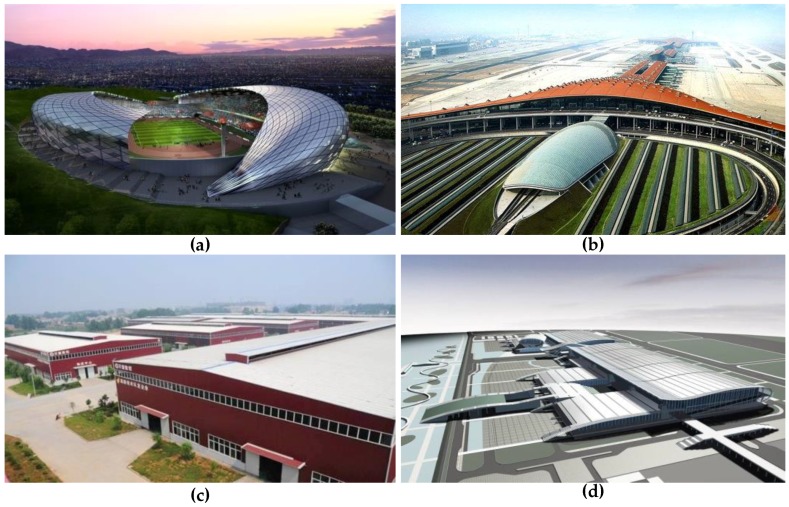
The application of metal roof in buildings: (**a**) Olympic Sports Center of Guiyang; (**b**) Beijing-Capital International Airport; (**c**) The production plant of Hubei Wanmeng CNC combination machine Limited company; (**d**) Guangzhou International Convention and Exhibition Center.

Metal roof sheathings are generally assembled from cold-formed steel with insulation, sound-absorbing insulation and waterproof materials. Due to their advantages of light weight, high strength, flexible design, unique shape and installation convenience, they are widely employed in large-span steel-structure buildings such as exhibition buildings, performing arts centers, transportation hubs, sports stadiums and production plants [1,2]. Figure 1 presents some typical metal roof systems on large span buildings. As an example, the maximum span of the Guangzhou International Convention and Exhibition Center is approximately 132.8 m and the covered area is about 500,000 m^2^.

**Figure 2:**
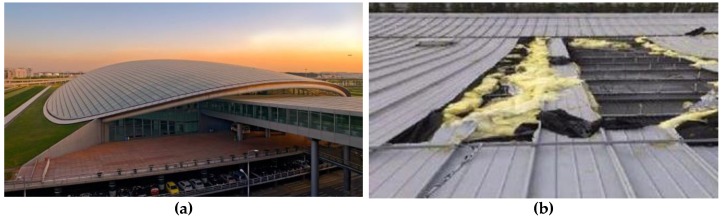
Wind-uplift destruction of metal roofs; (**a**) Beijing International Airport T3; (**b**) The metal roof panels of Beijing International Airport T3 were split by wind.

The main materials of metal roof systems include aluminum-magnesium-manganese (Al-Mn-Mg) alloy plate, titanium zinc plate and stainless steel with high strength and corrosion resistance. However, in their complex natural environment metal roof sheathings suffer the effects of wind, rain, snow, wind loads and thermal changes, leading to structural deformation, material fatigue, loose bolts and other possible failures. With the attenuation of wind-uplift resistance, the metal roof may be damaged even if the wind pressure does not exceed the design load [3]. In recent years, with the wide applications of metal roofs, several accidents were reported. For example, in March 2013, the metal roof of Beijing International Airport T3 was uplifted by strong winds, and the damaged area was about 200 square meters [4]. Figure 2 shows the large-scale broken area on the metal roof. On 18 July 2014, affected by the typhoon “Vimason”, the metal roof for the stadium of Qiongtai Normal University in HaiKou city (HaiNan province), suffered serious damage [5]. 

There is no doubt that the sustainability and resilience of large-scale civil infrastructure are of the utmost importance as they are closely related to people’s daily lives and social order. In recent years, some research progresses have been made on construction automation in civil engineering, intelligent sensing, and structural monitoring and health management [6,7,8,9,10]. La and Lim presented a mechatronic system design for an autonomous robotic system to inspect and evaluate bridge deck [7]. A project “Wi-Health” sponsored by the EC investigated the structural health monitoring based on multi-purpose wireless sensors network in which passive acoustic emission (AE) technology was combined with active long range ultrasonic (LRU) technology to monitor bridge structures [8].

At present, the safety detection of metal roof panels mainly relies on manual inspection, which unavoidably compromises the working efficiency and cannot diagnose and predict possible failures in time. Therefore, a real-time monitoring system is desirable to predict and estimate the potential serious deformation or damages so as to provide on-condition maintenance. When deformation or damages occur at the beginning, the deflection status of metal roof plates may change. Thus, the deflection distribution is a key characteristic and the essential issue of constructing monitoring system is to choose proper sensors and layout scheme.

When choosing detection technologies, several problems should be considered. For example, the metal roof system covers a large area, the working environment is complex and the installation space for measuring sensors is limited. Thus, it is desirable to adopt sensors that are easy to install, low-cost, and reliable under bad environmental conditions.

There have been many developments in detection approaches for curved surfaces, such as the three coordinate measuring machine, grating optical fiber [11], LASER-based methods, CT scanning methods, CCD camera detection methods [12], multimetric data (i.e., acceleration and strain) fusion [9] and so on. Although these approaches are suitable for some special situations, the characteristics of sensors such as continuous working hours, convenient installation, low power consumption and cost should be considered for actual monitoring of a metal roof. Therefore, we chose bend sensors that can be conveniently pasted on the surface of metal roof panels to detect their local deformation. Bend sensors [13] are sensitive to deformation based on optical fiber, conductive ink, electronic textiles, piezoelectric zinc oxide (ZnO) thin films [14], and tailored nanocomposite materials [15]. Bend sensors have been widely used in different fields, from simple angle measurements to the bending amount of human body joints [13,16,17,18] and shape-sensing in mechanical structures [13]. Due to their low-energy characteristics, bend sensors are suitable for a wide range of monitoring networks. Even so, it is often impractical in large-span buildings to cover all a roof surface by laying sensors in quantity. Thus, the deformation estimation from limited measurements is desired for whole roofs. Furthermore the potential fault prediction can be realized for metal roof systems. 

Deformation and stress distribution analysis of metal roofs can be achieved by analytical methods and numerical simulation methods. Metal roof panels can be modeled as a kind of elastic thin plate structure and the governing equations are solved with some simplified boundary conditions. It approach has distinct physical significance but low accuracy due to the structural or boundary simplifications, which is generally used for the preliminary design and analysis. The primary analytical methods include the Navier dual-trigonometric series, Levy solution, symplectic geometry, non-linear solutions and so on. At present, more valuable studies about analytical solutions of thin plate deformation have been developed considering different load types, structure characteristics and supporting modes. Nakai [19] presented an analytical method based entirely on elasto-plastic and large deflection theory to estimate the “critical strength” of various types of thin-walled steel frames. Yang [20] used the symplectic geometry method in a Hamilton system to derive the theoretical solution for the elastic cantilever rectangular thin plate. Batista [21] proposed a new analytical solution in the form of a Fourier series for the bending problem of a corner-supported rectangular thin plate under uniformly distributed load, in which σ-method was used to speed up the convergence rates. Zhong and Vinesh [22] adopted nonlinear large deformation theory to solve the bending of elastic thin plates. This method was valid to analyze nonlinear plates and could be extended to other boundary conditions. Van Gorder [23] discussed the linearization and construction of perturbation solutions for the Foppl-von Kármán equations, a set of non-linear partial differential equations describing the large deflections of thin flat plates. It could simplify the process of inverting the linear operators greatly so as to find the higher order terms comparing to numerical methods. Segovia [24] presented an approximated analytical Levy-type solution to analyze the vibration modal of a thin folded elastic structure. Apparently, these approaches are mostly obtained by extending or improving the abovementioned classical methods and suitable for some particular sorts of problems. Although sometimes the analytical method can get only an approximate solution for the elastic thin plate under specific loads and boundary conditions, it has faster convergence rate than numerical methods. Thus it is a rational choice in view of real-time estimation and on-line analysis needs.

When complex boundary conditions and loads have to be considered, numerical simulations such as finite-difference method and finite-element analysis (FEA) are generally suggested to obtain a detailed 3D deflection and stress field. For example, Song [25] explored the deflection and stress characteristics of standing seam metal roof panel under different negative pressure load by FEA software. Wang [26] applied a cube finite element model to analyze the piezothermoelasticity of an intelligent thin plate. Zhang [27] introduced numerical manifold method (NMM) to solve thin plate bending deformation problems. Turevsky [28] introduced an efficient numerical scheme to compute the topological sensitivity (TS) of arbitrary-shaped features in plate bending. Although numerical models can describe precisely boundary conditions and plates’ shapes, such as supporting modes, joints and reinforcing ribs, they are limited to off-line system analysis, design and optimization due to high computation consumption.

In this paper, we focus on real-time reconstruction of the deflection distribution for metal roof plates from a limited number of measured data of bend sensors, as the basis of potential fault prediction. The standing seam metal roof under wind pressure is modeled as a rectangular thin elastic plate. A fast analytic method based on Levy-type solution and the infinite series simplification is presented and then the model errors (the error between deflection of metal roof computed by reconstruction model and exact value) are analyzed. Finally, the corresponding simulations and experiments are conducted and measured data are compared with analytical solutions on the given detection points. The results show that the proposed reconstruction model can estimate the deflection distribution of metal plates in real-time.

## 2. Fast Reconstruction Model for Deflection Distribution of Metal Roof

### 2.1. A Fast Reconstruction Model for the Deflection Distribution of Metal Roof

**Figure 3:**
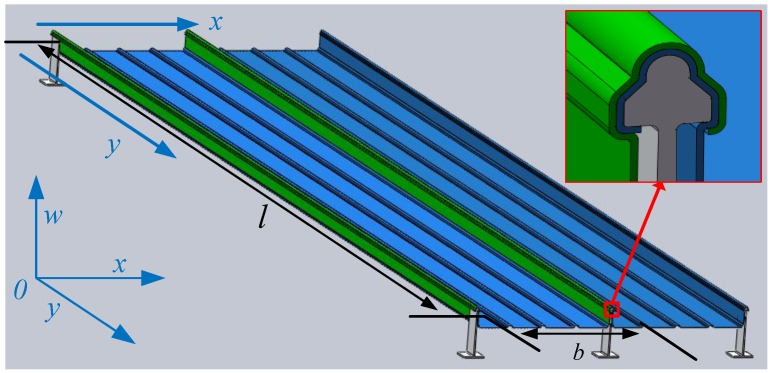
Metal roof model and its simplified diagram.

To establish the analytical model of metal roof deformation, a common standing seam metal roof is taken as an example. The entire metal roof is made up of many single metal roof sheathings locked together. As shown in Figure 3, two pieces of roof sheathing are assembled on the support. As the effect of the standing edges and buckling edges (green part in Figure 3), the two sides of *y* direction which form occlusal constraints with the metal holders can be seen as simply supported edges. The two sides in the *x* direction are equivalent to free edges because of the absence of constraints. 

Under the same weather condition, a single block of metal roof suffers a wind uplift equivalent to a uniform load. Therefore, the entire metal roof can be seen as a rectangular plate with two opposed simply supported edges and two other free opposed-edges. The control equations can be solved by external constraints and applied loads.

To analyze the deflection of the metal roof based on thin plate bending theory, the Kirchhoff hypothesis [29] is adopted as follows: (1) the thickness of the plate is much smaller than the horizontal and vertical dimensions; the density of the structure is constant throughout; (2) before the thin plate deforms, a straight line is perpendicular to the middle plane. It is still perpendicular to the middle plane after the thin plate deforms. The points on the middle surface have no horizontal displacement. 

#### 2.1.1. Governing Equations for Deflection Deformation of Thin Plate

**Figure 4:**
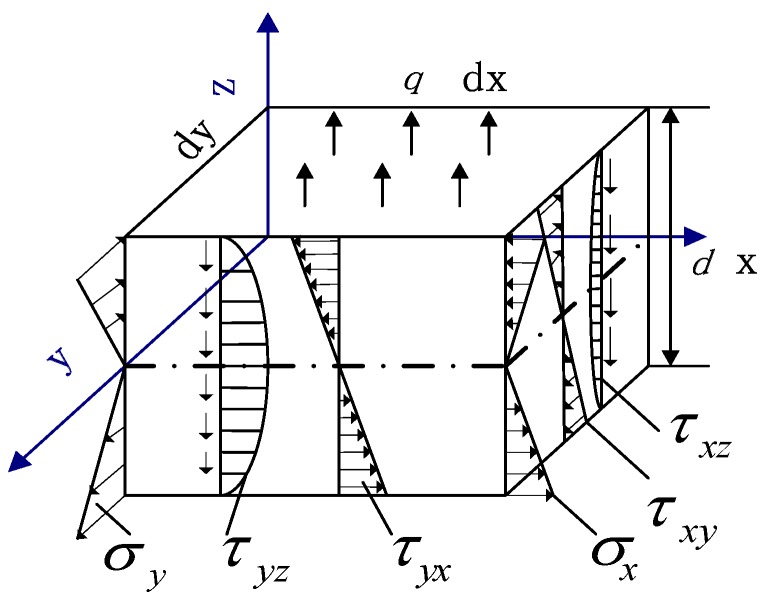
The stress distribution of hexahedral element.

As shown in Figure 4, we took a hexahedral element in the thin plate whose thickness is *d*. Assume that the upper surface is subjected to a uniform load *q*. Then its stress condition is analyzed.

From assumption (2), points in the middle have no displacement in the *x* and *y* direction, *u* and *v* are non-existent, and the displacements *u* and *v* whose distance were *z* from the middle could be obtained as:

$$u=-z\frac{\partial w}{\partial x},v=-z\frac{\partial w}{\partial y}.$$

According to the Hooker theorem, we can get the normal stress and shear stress as follows:

$$\sigma_{x}=-\frac{Ez}{1-u^{2}}[\frac{\partial^{2}w}{\partial x^{2}}+u\frac{\partial^{2}w}{\partial y^{2}}],\sigma_{y}=-\frac{Ez}{1-u^{2}}[\frac{\partial^{2}w}{\partial y^{2}}+u\frac{\partial^{2}w}{\partial x^{2}}],\tau_{xy}=-2Gz\frac{\partial^{2}w}{\partial x\partial y}\mathrm{and}G=\frac{E}{2(1+u)}.$$

The stress components $\tau_{zx},\tau_{zy}$ are determined by the following two equations:

$$\frac{\partial \sigma_{x}}{\partial x}+\frac{\partial \tau_{yz}}{\partial y}+\frac{\partial \tau_{zx}}{\partial z}=0,\frac{\partial \sigma_{y}}{\partial y}+\frac{\partial \tau_{zy}}{\partial z}+\frac{\partial \tau_{xy}}{\partial x}=0.$$

As shown in Figure 4, the normal stress and shear stress are linearly distributed in the z direction. The corresponding bending moments $M_{x}$, $M_{y}$, $M_{xy}$ and shearing forces $Q_{x}$, $Q_{y}$ can be obtained as:

(1) $$M_{x}=\int_{\frac{-d}{2}}^{\frac{d}{2}}\sigma_{x}zdz=-D(\frac{\partial^{2}w}{\partial x^{2}}+\nu \frac{\partial^{2}w}{\partial y^{2}})$$

(2) $$M_{y}=\int_{\frac{-d}{2}}^{\frac{d}{2}}\sigma_{y}zdz=-D(\frac{\partial^{2}w}{\partial y^{2}}+\nu \frac{\partial^{2}w}{\partial x^{2}})$$

(3) $$M_{xy}=-\int_{\frac{-d}{2}}^{\frac{d}{2}}\tau_{xy}zdz=D(1-\nu )\frac{\partial^{2}w}{\partial x\partial y}$$

(4) $$Q_{x}=\int_{-\frac{d}{2}}^{\frac{d}{2}}\tau_{xz}dz=-D(\frac{\partial^{3}w}{\partial x^{3}}+(2-\mu )\frac{\partial^{3}w}{\partial x\partial y^{2}})$$

(5) $$Q_{y}=\int_{-\frac{d}{2}}^{\frac{d}{2}}\tau_{yz}dz=-D(\frac{\partial^{3}w}{\partial y^{3}}+(2-\mu )\frac{\partial^{3}w}{\partial y\partial x^{2}})$$

The governing equations of the bending plate can be obtained based on the force balance as follows [30]:

(6) $$\frac{\partial^{4}w}{\partial x^{4}}+2\frac{\partial^{4}w}{\partial x^{2}\partial y^{2}}+\frac{\partial^{4}w}{\partial y^{4}}=\frac{q}{D},$$

where $D=Ed^{3}/12(1-\mu^{2})$ is the flexural rigidity of the plate, *E* is Young’s modulus of the plate, *µ* is Poisson’s ratio, *w* is the vertical deflection and *q* is the external uniform load.

As shown in Figure 3, the metal roof can be simplified as a thin plate with two opposite simply-supported edges, two free edges and subjected to a uniformly distributed load.

The boundary conditions are as follows:

I Free edges (*y* = 0, *y* = *l*): the bending moment and shear force are zero, and formulated as:
  $$D(\frac{\partial^{2}w}{\partial x^{2}}+\mu \frac{\partial^{2}w}{\partial y^{2}})=0,D(\frac{\partial^{3}w}{\partial x^{3}}+(2-\mu )\frac{\partial^{3}w}{\partial x\partial y^{2}})=0.$$
II Supported Edges (*x* = 0, *x* = *b*): the deflection and bending moment are zero:
  $$D(\frac{\partial^{2}w}{\partial x^{2}}+\mu \frac{\partial^{2}w}{\partial y^{2}})=0.$$

The superposition method is used to solve the above problems. They are treated as the problem that a rectangular thin plate with the two opposite free sides and the other two opposite sides simply supported subjected to a uniform load *q*. The two parts of superposition are taken as follows:

(a) A thin rectangular plate with four sides simply supported, and subjected to uniform load *q*.
(b) A rectangular plate with two sides *x* = 0 and *x* = *b* seen as simply supported sides, and the other sides *y* = 0 and *y* = *l* seen as generalized simply supported edges.

#### 2.1.2. Deflection Calculation of Part I

The deflection of this part is assumed as $w_{a}(x,y)$, and the expression of its double triangle series is:

(7) $$w_{a}(x,y)=\sum_{m=1}^{\infty }\sum_{n=1}^{\infty }\alpha_{mn}\sin\frac{m\pi x}{b}\sin\frac{n\pi y}{l},$$

The double triangle series expansion of the uniform load *q* is:

(8) $$q=\sum_{m=1}^{\infty }\sum_{n=1}^{\infty }A_{mn}\sin\frac{m\pi x}{b}\sin\frac{n\pi y}{l},$$

where $A_{mn}=\frac{16q}{\pi^{2}}\cdot \frac{1}{mn}$ (*m*, *n* = 1, 3, 5…), and $A_{mn}=0$ (*m* and *n* is even).

Equations (7) and (8) are substituted into Equation (6), then both sides of the equation coefficients are compared and the final deflection equation is:

(9) $$w_{a}(x,y)=\frac{16q}{\pi^{6}D}\sum_{\begin{array}{l} m=2i+1\\ i=0 \end{array}}^{\infty }\frac{1}{m^{5}}\sin\frac{m\pi x}{b}\sum_{\begin{array}{l} n=2j+1\\ j=0 \end{array}}^{\infty }\frac{l^{4}}{n{\left(n^{2}/m^{2}+l^{2}/b^{2}\right)}^{2}}\sin\frac{n\pi y}{l}.$$

Equation (9) is double trigonometric series, so to speed up the convergence rate, the sum of series containing *y* is calculated, and the Levy-type solution [31] is adopted and Equation (9) can be rewritten as:

(10) $$w_{a}(x,y)=\frac{4qb^{4}}{\pi^{5}D}\sum_{\begin{array}{l} m=2j+1\\ j=0 \end{array}}^{\infty }\frac{1}{m^{5}}g_{m}(x)\cdot f_{m}(y),$$

where:

(11) $$g_{m}(x)=\sin\frac{m\pi x}{b},$$

(12) $$f_{m}(y)=[\lambda_{m}(y-\frac{l}{2})\sinh\lambda_{m}(y-\frac{l}{2})-(2+\frac{\lambda_{m}l}{2}\tanh\frac{\lambda_{m}l}{2})\cosh\lambda_{m}(y-\frac{l}{2})]\frac{1}{2\cosh(\lambda_{m}l/2)}+1,$$

and $\lambda_{m}=m\pi /b$ (*m* = 1, 3, 5…).

By investigating the convergence rate of the function series, we find that the convergence of the function series depends mainly on the first item. Take the first term of series, i.e.:

(13) $$w_{a}^{\left(1\right)}(x,y)=\frac{4qb^{4}}{\pi^{5}D}g_{1}(x)f_{1}(y),$$

and the remainder is:

(14) $$R_{a}=\frac{4qb^{4}}{\pi^{5}D}\cdot \sum_{\begin{array}{l} m=2i+1\\ i=1 \end{array}}^{\infty }\frac{1}{m^{5}}g_{m}(x)\cdot f_{m}(y).$$

Refer to Appendix A. It can be proved that:

(15) $$\left|\frac{R_{a}}{w_{a}^{\left(1\right)}(x,y)}\right|<<1,$$

thus, it is reasonable to take the first term of series to approximate $w_{a}$ so as to decrease computing time. Thus we get:

(16) $${\tilde{w}}_{a}(x,y)=w_{a}^{\left(1\right)}(x,y)=\frac{4qb^{4}}{\pi^{5}D}g_{1}\left(x\right)f_{1}(y).$$

#### 2.1.3. Deflection Calculation of Part II

The deflection of part II is $w_{b}$.Using the same methods as mentioned in Section 2.1.1 to analyze the deflection of part II, the following result can be obtained:

(17) $$w_{b}(x,y)=\frac{qb^{4}}{\pi^{5}D}\sum_{\begin{array}{l} n=2j+1\\ j=0 \end{array}}^{\infty }\frac{1}{n^{5}}g_{n}(x)\cdot h_{n}(y)$$

where $\lambda_{n}=n\pi /b$ and

(18) $$h_{n}(y)=(a_{1}+c_{1}\lambda_{n}y)\cdot \sinh\lambda_{n}y+(a_{2}+c_{2}\lambda_{n}y)\cdot \cosh\lambda_{n}y.$$

To obtain $h_{n}(y)$, boundary conditions are used to solve the problem:

(1) Moment of the two generalized simply supported edges is zero, i.e., $M=0{|}_{y=0,l}$

(2) Deflection of the two generalized simply supported sides is assumed, i.e., $w=\alpha_{0}\sin(\beta x){|}_{y=0,l}$, and $h_{n}(y)$ can be given:
  (19) $$h_{n}(y)=\alpha_{0}\left\{\frac{\cosh\lambda_{n}l-1}{\sinh\lambda_{n}l}[(\frac{\lambda_{n}l}{\sinh\lambda_{n}l}-\frac{1}{1-\mu })\sinh\lambda_{n}y+\lambda_{n}y\cosh\lambda_{n}y]+\frac{1}{1-\mu }\cosh\lambda_{n}y-\lambda_{n}y\sinh\lambda_{n}y\right\}$$

According to the boundary conditions, $\alpha_{0}$ is calculated:

(20) $$\alpha_{0}=\frac{2\tanh\frac{\lambda_{n}l}{2}[(4-\mu )-(1-\mu )\frac{\lambda_{n}l}{2\cosh(\lambda_{n}l/2)}]}{{\left(1-\mu \right)}^{2}(\cosh\lambda_{n}l-1)(\frac{3+\mu }{1-\mu }-\frac{\lambda_{n}l}{\sinh\lambda_{n}l})}.$$

The first term of Equation (12) is represented as:

(21) $$w_{b}^{\left(1\right)}(x,y)=\frac{qb^{4}}{\pi^{5}D}h_{1}(y)\cdot g_{1}(x),$$

and the remainder is:

(22) $$R_{b}=\frac{qb^{4}}{\pi^{5}D}\cdot \sum_{\begin{array}{l} n=2j+1\\ j=1 \end{array}}^{\infty }\frac{1}{n^{5}}g_{n}(x)\cdot h_{n}(y).$$

It can be proved that (Appendix A):

(23) $$\left|\frac{R_{b}}{w_{b}^{\left(1\right)}(x,y)}\right|<<1.$$

Then the estimated formula is obtained as:

(24) $${\tilde{w}}_{b}(x,y)=w_{b}^{\left(1\right)}(x,y)=\frac{qb^{4}}{\pi^{5}D}h_{1}(y)\cdot g_{1}(x).$$

Superimposing the deflection of two parts gives the deflection of the coordinate (x,y) on metal roof:

(25) $$w(x,y)=w_{a}(x,y)+w_{b}(x,y)=\frac{qb^{4}}{\pi^{5}D}\sum_{\begin{array}{l} m=2i+1\\ i=0 \end{array}}^{\infty }\frac{1}{m^{5}}g_{m}(x)\cdot \left[4f_{m}(y)+h_{m}(y)\right].$$

Take the first term of series as the estimated deflection of the metal roof, i.e.:

(26) $$\tilde{w}(x,y)=w^{\left(1\right)}(x,y)=q\cdot g_{1}(x)\cdot \frac{b^{4}}{\pi^{5}D}(4f_{1}(y)+h_{1}(y)).$$

When the uniform load *q* is unknown, the deflection of installation location $\left(x_{0},y_{0}\right)$ can be measured by a bend sensor, and the value is $w_{0}(x_{0},y_{0})$. Substituting $w_{0}(x_{0},y_{0})$ and (x,y) into Equation (26) yields the deflection of any point on the metal-roof:

(27) $$\tilde{w}(w_{0},x,y)=\frac{w_{0}(x_{0},y_{0})}{g_{1}(x_{0})\cdot (4f_{1}(y_{0})+h_{1}(y_{0}))}\cdot g{\left(x\right)}_{1}\cdot (4f_{1}(y)+h_{1}(y)).$$

### 2.2. Analysis of Model Error 

Based on the above analysis, the exact deflection of point (x,y) on a metal roof is represented in Equation (25) and the estimated value is in Equation (26). The remainder is:

(28) $$R=R_{a}+R_{b}=\frac{qb^{4}}{\pi^{5}D}\sum_{\begin{array}{l} m=2i+1\\ i=1 \end{array}}^{\infty }\frac{1}{m^{5}}g_{m}(x)\cdot \left[4f_{m}(y)+h_{m}(y)\right].$$

The relative error between w(x,y) and $\tilde{w}(x,y)$ is:

(29) $$\varepsilon =\left|\frac{R}{w(x,y)}\right|.$$

According to Equations (11), (12), (19), (23) and (27), to calculate the value of ε, the value of x,y,l,b should be determined. We choose 65/400 straight side whipstitch plate type roof panel [32] for experiment. Its properties are shown in Table 1.

The value scope of *x* is 0~400 mm, the value scope of *y* is 0~1700 mm and l/b=17/4. According to Equations (15) and (23), we can obtain:

(30) $$\varepsilon =\left|\frac{R_{a}+R_{b}}{w_{a}(x,y)+w_{b}(x,y)}\right|\approx \left|\frac{R_{a}+R_{b}}{{\tilde{w}}_{a}(x,y)+{\tilde{w}}_{b}(x,y)}\right|\leq \left|\frac{R_{a}}{{\tilde{w}}_{a}(x,y)}\right|+\left|\frac{R_{b}}{{\tilde{w}}_{b}(x,y)}\right|.$$

**Figure A5:**
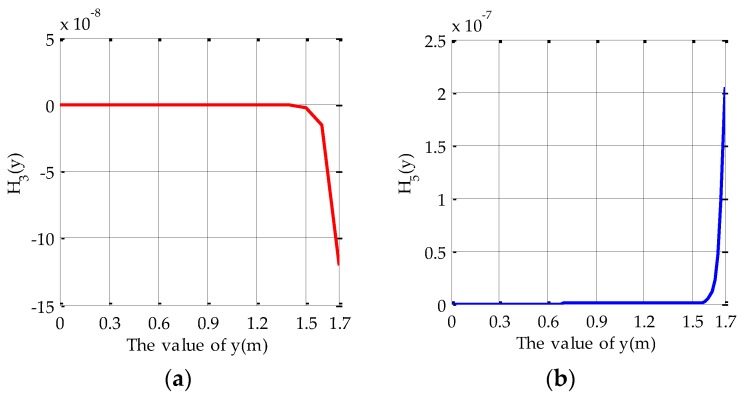

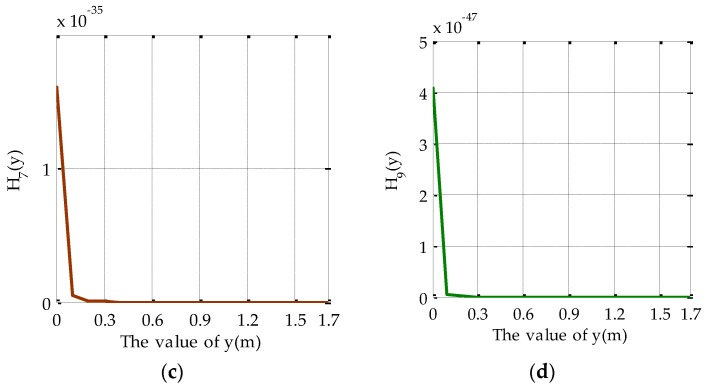
The value of $H_{n}(y)$: (**a**) *n* = 3; (**b**) *n* = 5; (**c**) *n* = 7; (**d**) *n* = 9.

According to Figure A5 and Equation (A9) of Appendix B, it can be obtained that the value of $\left|\frac{R_{b}}{w_{b}^{\left(1\right)}(x,y)}\right|$ is approximately 0. And according to Equation (A8), the maximum relative error is:

(31) $$\varepsilon \leq \left|\frac{R_{a}}{{\tilde{w}}_{a}(x,y)}\right|+\left|\frac{R_{b}}{{\tilde{w}}_{b}(x,y)}\right|\approx \left|(7*\zeta (3))/8-1\right|+\left|\frac{\pi^{4}}{48}-2\right|\times 10^{-7}\approx 0.0518.$$

## 3. Simulations and Experiments

In order to verify the proposed fast reconstruction model of deflection distribution for metal roofs, the corresponding simulations and experiments are carried out and discussed in this section. Firstly, we performed a real value simulation (using the practical size and properties of metal roof sheathings). We simulated the deflection distributions of a metal roof panel under uniform load by using the proposed analytical method and the FEA method. The results and computation time are analyzed to validate the accuracy and rapidity of the fast reconstruction model. Next, an experimental standing seam metal roof platform is set up according to the metal roofs used in practical engineering applications and the bend sensors are calibrated. Finally, a series of experiments are designed and implemented to verify the practicability of online monitoring scheme of metal roof deformation based on bend sensors and the fast reconstruction model.

### 3.1. Simulation and Discussions 

In the real-size simulation experiments, the 65/400 straight side whipstitch plate type roof panel is selected as test sample and its properties are shown in Table 1. The mathematical software MATLAB 2012 is used to compute the reconstruction model of this metal roof panel in which 20 × 85 points ($\vec{x}\times \vec{y}$) are picked uniformly on the whole metal roof panel for deflection calculation. Meanwhile, we use the FEA software COMSOL 5.3b (COMSOL Inc, Stockholm, Sweden) to simulate the deflection distribution of metal roof panels which were meshed into 64 thousand elements on the same computer.

**Figure 5:**
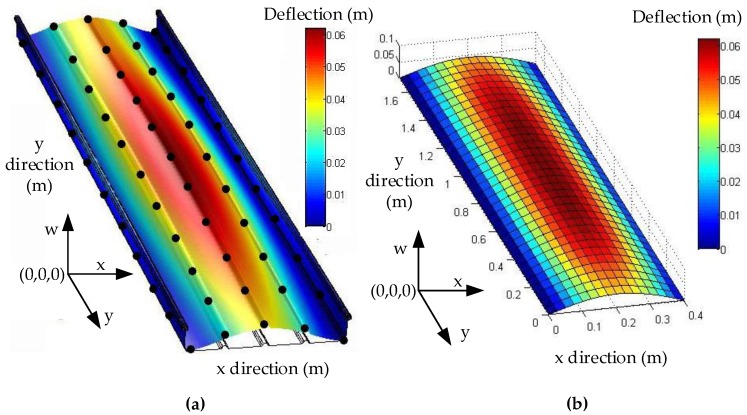
Comparison of two simulation methods; (**a**) Simulation results in COMSOL; (**b**) Simulation results in MATLAB.

**Figure 6:**
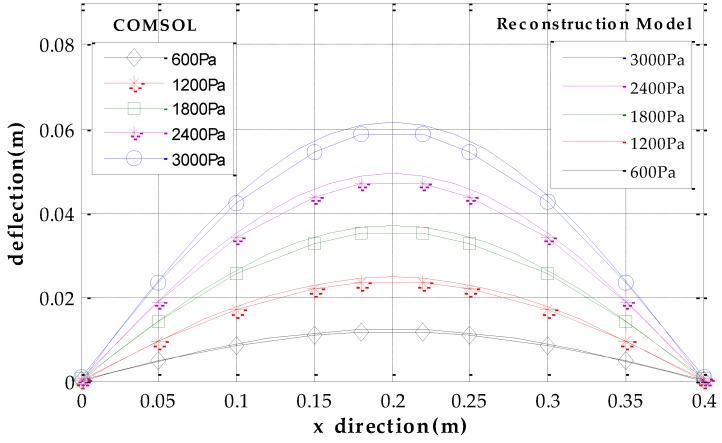
Deflection under different pressure in *x* direction (*y* = 0.85 m).

**Figure 7:**
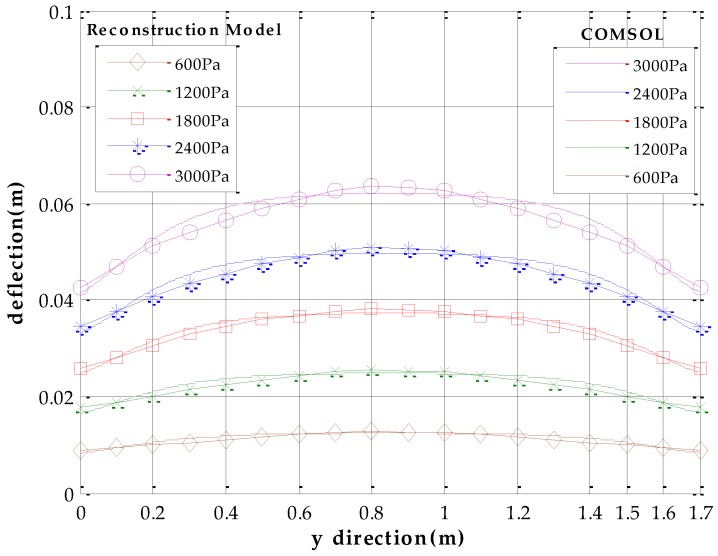
Deflection under different pressure in *y* direction (*x* = 0.18 m).

**Figure 8:**
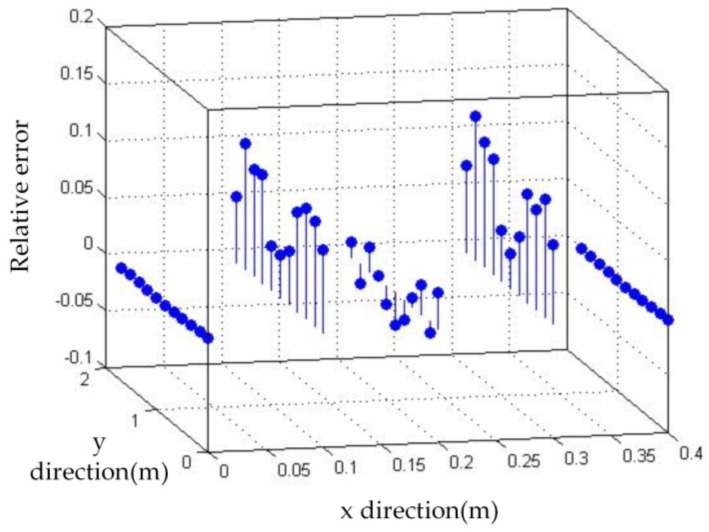
Relative error of the two methods.

We use software MATLAB and COMSOL to simulate the deformation of metal roof panel with loads from 0~3000 Pa in steps of 200 Pa. When the equivalent uniform load *q* imposed on metal panel is 3000 Pa [33], Figure 5 shows the simulation results of the FEA model in COMSOL and the fast reconstruction model in MATLAB. It can be seen that the deflection distributions are roughly consistent in both sets of results. Furthermore, we take the COMSOL data of picked points (five lines and eleven rows of points on the plate, i.e., black points in Figure 5a) as the reference base to examine the relative error of the analytical model. Figure 6 shows deflection under different loads in the *x* direction (*y* = 0.85 m), and Figure 7 shows deflection under different loads in *y* direction (*x* = 0.18 m, avoid the location of the stiffeners). From Figure 6 and Figure 7, it can be seen that the results of reconstruction model are consistent with the simulation results in COMSOL. The error data are shown in Figure 8. The absolute error is mm-level, and maximum relative errors occur on the stiffener locations where the stiffeners are modeled in COMSOL simulation but simplified through assumptions in the analytical model. We are more interested in the maximum deflection and the deflection at the boundaries. The maximum error on these key positions is about 2 mm and percentage error is 2% where our applicable percentage error is 5%, so the fast analytical model is applicable to deflection calculations of metal roof panel.

In the same hardware platform, the calculation time of MATLAB is about 20 ms, whereas that of COMSOL is approximately 30 min. Obviously, the fast reconstruction model saves computer hardware resources and takes less time to compute the metal roof deflection with comparable precision. Moreover, according to the measured circuit design features and sampling period we have known that the general time of collecting and transmitting data is about 15 ms, thus the fast reconstruction model based on measured data can meet the requirements of real-time monitoring.

### 3.2. Experimental Platform

**Figure 9:**
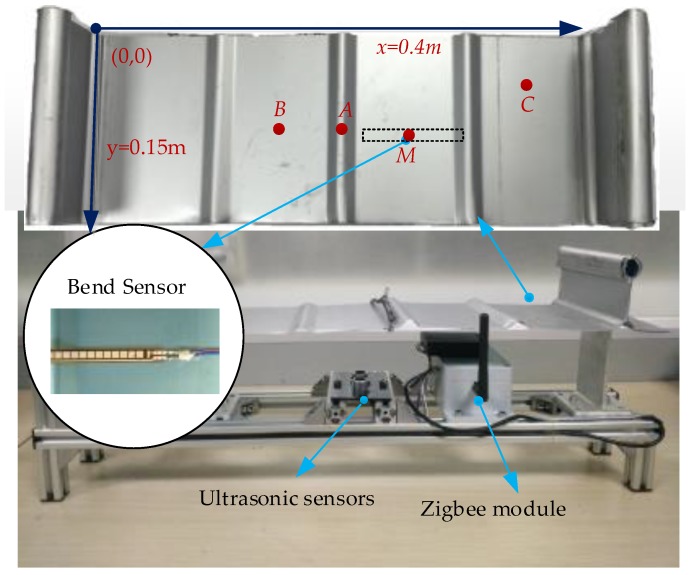
Standing seam metal experimental table.

In order to facilitate the experiment, a piece of Al-Mg-Mn metal roof panel is incised to set up experimental platform. The experimental platform is structured with 65/400 type (Al-Mg-Mn alloy) standing seam plate, bracket, bend sensor, ultrasonic module, Zigbee module and power module, as shown in Figure 9. The reference point of the bend sensor is M and its coordinate is (250,70) (mm). The given measurement points of the ultrasonic module are A(200,75),B(150,75),C(350,50) (mm) on the metal plate. The ultrasonic module is fixed on the bracket, whose upside is against the given points. The measured deflections of all sensors are transmitted by Zigbee modules, to PC for further analysis. The material properties of standing seam metal roof are given in Table 2.

We adopted the FS-L-0112-103-ST flex sensor (Spectra System, Salt Lake, UT, USA) in the experiment [13]. It is cheap, easy to integrate and requires no complicated signal processing. According to the manual, its continuous power rating is 0.50 W; its life cycle is more than one million uses; the sensitivity can be adjusted by circuit design [34].

The resistance changes with the bending status. Additionally, the working temperature range of the sensor is wide and not affected by the humidity. It can adapt to the complex and changeable situation of the temperature and humidity on the top of buildings. As shown in Figure 9, the bend sensor is stuck on the underside of the metal-roof plate. The bending status of the sensor changes with any deformation of the metal roof, which leads to a corresponding resistance change. 

**Figure 10:**
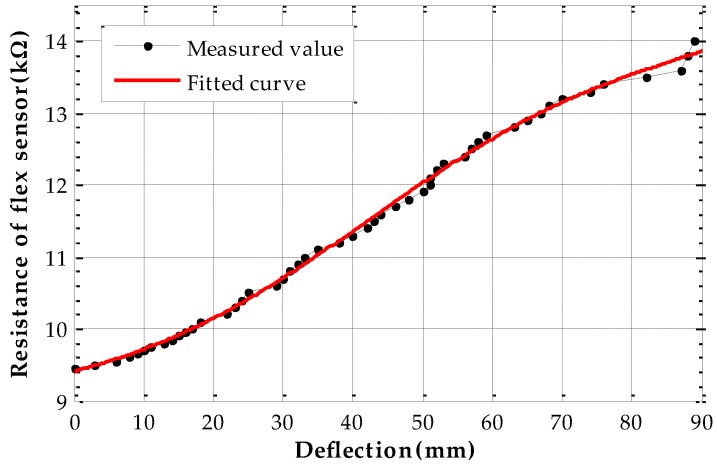
Flex sensor resistance-deflection curve fitting.

The bend sensor is calibrated on the experimental platform firstly. In the calibration experiments, we apply uniformly negative loads on the metal roof to make the deflection of the measuring point vary from 0 to 90 mm. Every deflection state lasts 60 s and the resistances of the flex sensor are measured with 1 Hz sampling frequency, but only stable values are recorded as calibration data after filtering. Then we use the data to fit deflection-resistance curve as shown in Figure 10 and the relationship of deflection-resistance value is assumed as:

(32) $$w(r)=a_{0}\times r^{5}+a_{1}\times r^{4}+a_{2}\times r^{3}+a_{3}\times r^{2}+a_{4}\times r.$$

The coefficients are given as:

$$a_{0}=-0.02125,a_{1}=1.091,a_{2}=-20.83,a_{3}=176.3,a_{4}=-557.2$$

**Figure 11:**
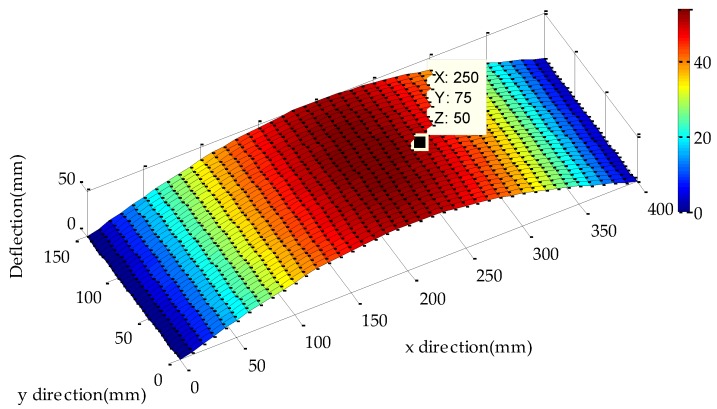
Deformation of entire metal roof.

When the deflection measured by bend sensor is 50 mm, 40 × 30 points ($\vec{x}\times \vec{y}$) on the experimental metal roof are taken to calculate their deflections according to Equation (22), and the deflection distribution of entire metal roof is plotted as shown in Figure 11. 

### 3.3. Experimental Results and Discussion

Imposed stepped reverse loads on the metal roof make the deflection of point *M* measured by the bend sensor change from 0 to 60 mm in 10 mm steps, approximately. Simultaneously, we report the deflections of points A, B and C from ultrasonic sensors. Substituting the measured values of the M point into Equation (22) we can get the theoretical deflection values of points A, B and C. The measured and estimated values of points A, B and C are summarized in Table 3.

**Figure 12:**
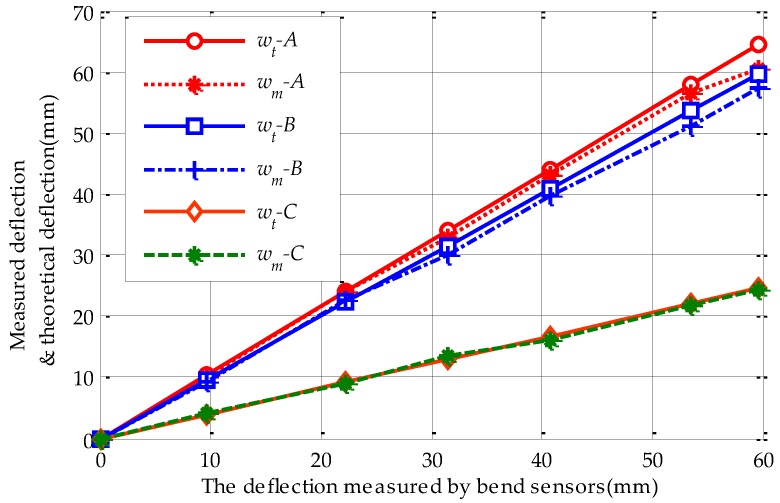
Theoretical and measured deflection values of different points.

The relationships between theoretical and measured values of points A, B and C are shown as Figure 12, where $w_{t}-A$, $w_{t}-B$ and $w_{t}-C$ represent the theoretical value of points A, B and C; $w_{m}-A$, $w_{m}-B$ and $w_{m}-C$ represent the measured values at points A, B and C.

From Table 3 and Figure 12, the estimated deflections calculated by the reconstruction model agree well with the measured data on the whole. The maximum relative error (7.1%) occurs at point A which is on the metal roof stiffener; this is because the stiffener can increase the strength of the plate and reduce the deformation of the metal roof. Additionally, in lines A and B, there is a slight difference between the measured data and theoretical calculation at higher deflection values. The possible reason is that the bend deformation of the metal roof panel may be out of the elastic deformation range at higher deflection values in the actual experiments. Meanwhile, the bend deformation is assumed elastic in the whole deformation process in the reconstruction model. Its effect can be neglected since the difference is small and not significant for damage estimation.

## 4. Conclusions

A novel deformation-monitoring scheme for metal roof sheathing based on bend sensors has been presented. A fast reconstruction model is established to estimate the deflection distribution of whole roof panels based on limit measured data. The superposition method and Levy solution are adopted to obtain an analytical model with quick convergence by simplifying infinite series. Then, the simulation study of the real-size metal roof panel shows that the results of the proposed method agree well with those of the COMSOL model but require much less computation time. Finally, an experimental investigation is conducted to validate this scheme. The results confirm that the fast reconstruction model can estimate the deflection distribution of the whole metal plate in terms of computational accuracy and time, which provides a data basis for the online health monitoring of large-span metal roof sheathing systems which will be further studied in our future work.

## Figures and Tables

**Table 1:** Material properties of 65/400 standing seam metal roof.

Material	Length *l* (mm)	Width *b* (mm)	Thickness *d* (mm)	Poisson Ratio	Young Modulus (Pa) *E* (N/m^2^)
AA3004 Al-Mg-Mn alloy	1700	400	1	0.3	7 × 10^10^

**Table 2:** Material properties of standing seam metal roof.

Roof Material	Lock Edge	Free Edge	Thickness	Poisson Ratio	Young’s Modulus
	*l* (mm)	*b* (mm)	*d* (mm)	*μ*	*E* (N/m^2^)
AA3004 Al-Mg-Mn alloy	150	400	1	0.3	7 × 10^10^

**Table 3:** Comparison of measured and theoretical values of deflection of metal roof.

Measured Points		Deflection (mm)							Max Relative Error (%)
M		0	9.6	22.3	31.5	40.7	53.5	59.6	
A	TV	0	10.4	24.1	34.1	42.7	57.9	64.5	7.1%
	MV	0	10.2	22.5	33.0	42.0	54.6	62.0	
B	TV	0	9.6	22.3	31.5	40.7	53.5	59.6	3.6%
	MV	0	9.3	21.5	32.7	42.1	53.8	57.5	
C	TV	0	3.9	9.2	13.0	20.1	22.0	24.6	5.3%
	TV	0	3.8	8.9	12.5	19.1	21.5	23.9	

Note: TV represents theoretical value; and MV represents measured value.

**Figure A1:**
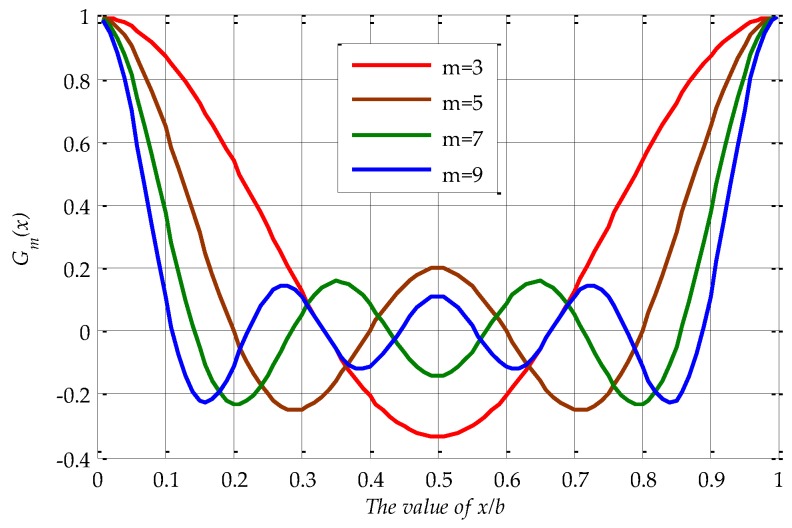
The value of $G_{m}(x)$.

**Figure A2:**
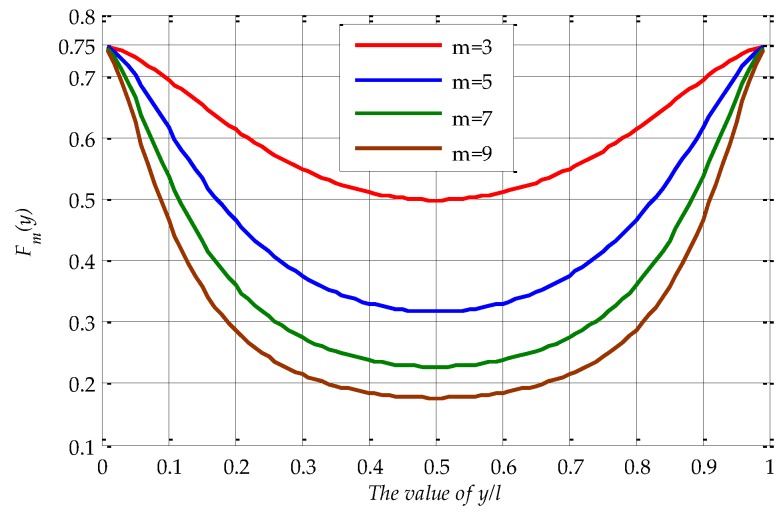
The value of $F_{m}(y)$.

**Figure A3:**
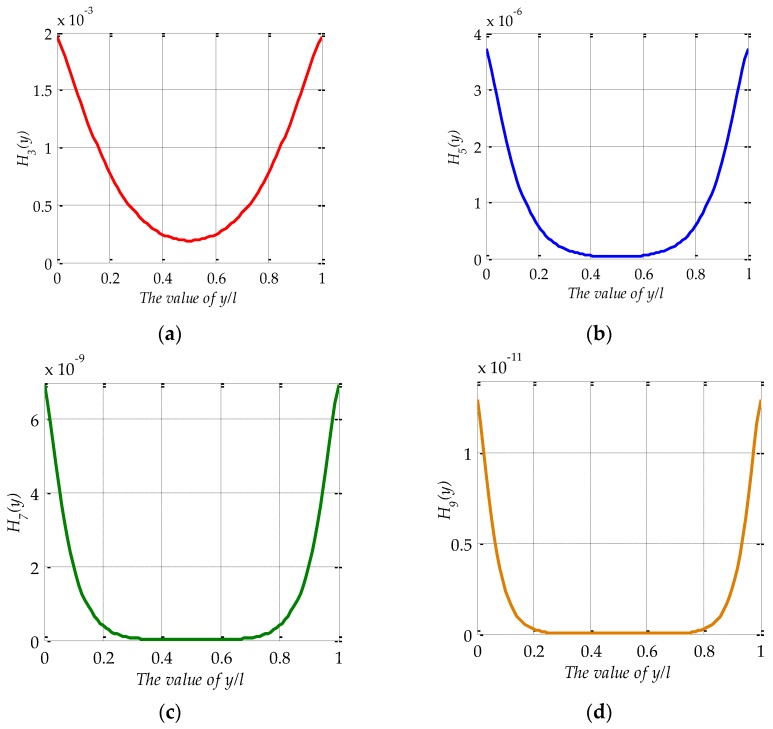
The value of $H_{n}(y)$: (**a**) *n* = 3; (**b**) *n* = 5; (**c**) *n* = 7; (**d**) *n* = 9.

**Figure A4:**
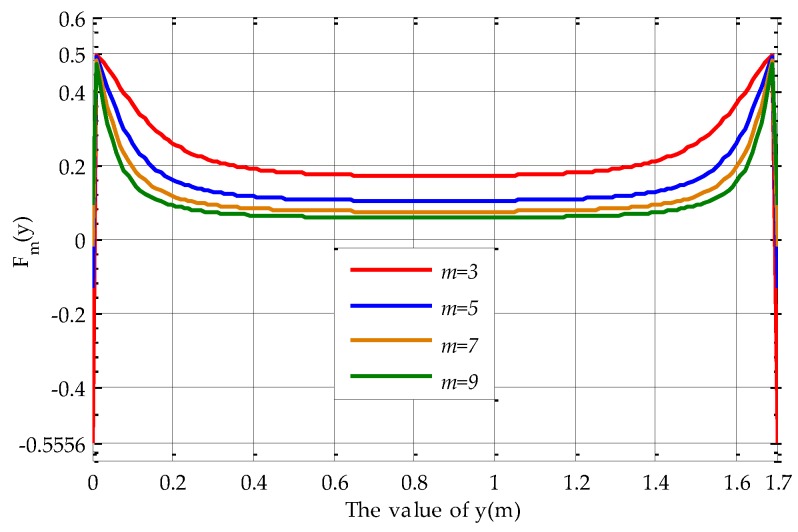
The value of $F_{m}(y)$.

## Appendix A

1. According to Equations (8) and (9), we can get that:

(A1) $$\left|\frac{R_{a}}{w_{a}^{\left(1\right)}(x,y)}\right|=\left|\frac{\sum_{\begin{array}{l} m=2i+1\\ i=1 \end{array}}^{\infty }\frac{1}{m^{5}}g_{m}(x)\cdot f_{m}(y)}{g_{1}(x)f_{1}(y)}\right|.$$

According to Equations (6) and (7), to calculate the value of $\left|\frac{R_{a}}{w_{a}^{\left(1\right)}(x,y)}\right|$, the value of x,y,b,l should be given. Obviously, the value range of *x* is from 0 to *b*, and the value range of *y* is from 0 to *l*. In actual use, the width of standing seam metal roof panel is 300 mm~500 mm, and the max length is up to 15,000 mm. Therefore, 1<l/b<50 and here we consider extreme situation in order to compute maximum truncation error, i.e., l/b=1. Define $G_{m}(x)=\frac{1}{m}\left|\frac{g_{m}(x)}{g_{1}(x)}\right|$ and $F_{m}(y)=\frac{1}{2m}\left|\frac{f_{m}(y)}{f_{1}(y)}\right|$. The value curves of $G_{m}(x)$ for *m* = 3, 5, 7, 9 are shown as Figure A1.

From Figure A1, we can infer that:

(A2) $$G_{m}(x)=\frac{1}{m}\cdot \left|\frac{g_{m}(x)}{g_{1}(x)}\right|=\frac{1}{m}\left|\frac{\sin\frac{m\pi x}{b}}{\sin\frac{\pi x}{b}}\right|\leq 1(m=2i+1\;\mathrm{and}\;i=1,2,\cdots \infty ).$$

The value of $F_{m}(y)$ is shown in Figure A2.

and it can be obtained that:

(A3) $$F_{m}(y)=\frac{1}{2m}\left|\frac{f_{m}(y)}{f_{1}(y)}\right|\leq 0.75.$$

So that:

(A4) $$\left|\frac{R_{a}}{w_{a}^{\left(1\right)}(x,y)}\right|=\left|\frac{\sum_{\begin{array}{l} m=2i+1\\ i=1 \end{array}}^{\infty }\frac{1}{m^{5}}g_{m}(x)\cdot f_{m}(y)}{g_{1}(x)f_{1}(y)}\right|=\left|\sum_{\begin{array}{l} m=2i+1\\ i=1 \end{array}}^{\infty }\frac{2}{m^{3}}G_{m}(x)\cdot F_{m}(y)\right|<\left|\sum_{\begin{array}{l} m=2i+1\\ i=1 \end{array}}^{\infty }\frac{1.5}{m^{3}}\right|=\left|\frac{21\times \zeta (3)}{16}-\frac{3}{2}\right|\approx 0.0777<<1,$$

where ζ(3) is three-order Riemann function.

2. According to Equation (14) and defining $H_{n}(y)=\left|\frac{h_{n}(y)}{h_{1}(y)}\right|$, Figure A3 shows the value of $H_{n}(y)$ with different *n*, it can be seen that the value of $H_{n}(y)$ tends to zero rapidly as *n* increases. Thus:

(A5) $$H_{n}(y)=\left|\frac{h_{n}(y)}{h_{1}(y)}\right|<<1.$$

According to Equations (14)–(16), it can be got that:

(A6) $$\left|\frac{R_{b}}{w_{b}^{\left(1\right)}(x,y)}\right|=\left|\frac{\sum_{\begin{array}{l} n=2j+1\\ j=1 \end{array}}^{\infty }\frac{1}{n^{5}}g_{n}(x)\cdot h_{n}(y)}{g_{1}(x)f_{1}(y)}\right|=\left|\sum_{\begin{array}{l} n=2j+1\\ j=1 \end{array}}^{\infty }\frac{1}{n^{4}}G_{n}(x)\cdot H_{n}(y)\right|\leq \left|\sum_{\begin{array}{l} n=2j+1\\ j=1 \end{array}}^{\infty }\frac{1}{n^{4}}\cdot H_{n}(y)\right|=\left|\frac{\pi^{4}}{96}-1\right|\cdot \left|H_{n}(y)\right|<<1$$

## Appendix B

We chose a 65/400 straight side whipstitch plate type roof panel as analysis object. Its properties are listed in Table 1. The value range of *x* is from 0 to 0.4 m, the value range of *y* is from 0 to 1.7 m, and l/b=17/4. According to Equation (A1), it can be obtained when $G_{m}(x)\leq 1$.

The value of $F_{m}(y)$ is shown as Figure A4.

and it can be obtained that:

(A7) $$F_{m}(y)=\frac{1}{2m}\left|\frac{f_{m}(y)}{f_{1}(y)}\right|\leq 0.5,$$

so that:

(A8) $$\left|\frac{R_{a}}{w_{a}^{\left(1\right)}(x,y)}\right|=\left|\frac{\sum_{\begin{array}{l} m=2i+1\\ i=1 \end{array}}^{\infty }\frac{1}{m^{5}}g_{m}(x)\cdot f_{m}(y)}{g_{1}(x)f_{1}(y)}\right|=\left|\sum_{\begin{array}{l} m=2i+1\\ i=1 \end{array}}^{\infty }\frac{2}{m^{3}}G_{m}(x)\cdot F_{m}(y)\right|<\left|\sum_{\begin{array}{l} m=2i+1\\ i=1 \end{array}}^{\infty }\frac{1}{m^{3}}\right|=\left|\frac{7*\zeta (3)}{8}-1\right|\approx 0.0518.$$

The value of $H_{n}(y)$ is shown as Figure A5.

According to Equations (A2) and (A3), and Figure A5, it can be obtained that:

(A9) $$\left|\frac{R_{b}}{w_{b}^{\left(1\right)}(x,y)}\right|=\left|\frac{\sum_{\begin{array}{l} n=2j+1\\ j=1 \end{array}}^{\infty }\frac{1}{n^{5}}g_{n}(x)\cdot h_{n}(y)}{g_{1}(x)f_{1}(y)}\right|=\left|\sum_{\begin{array}{l} n=2j+1\\ j=1 \end{array}}^{\infty }\frac{1}{n^{4}}G_{n}(x)\cdot H_{n}(y)\right|\leq \left|\sum_{\begin{array}{l} n=2j+1\\ j=1 \end{array}}^{\infty }\frac{1}{n^{4}}\cdot H_{n}(y)\right|\leq \left|\frac{\pi^{4}}{48}-2\right|\times 10^{-7}.$$
